# Outcomes and prognostic factors in dogs with subcutaneous and intramuscular hemangiosarcoma treated with hypofractionated radiation therapy and chemotherapy

**DOI:** 10.3389/fvets.2026.1925779

**Published:** 2026-10-06

**Authors:** Ana Paula Rebelo, Adriana Furtado, Rance Sellon, Gregory D. Roberts, Janean Fidel

**Affiliations:** Department of Veterinary Clinical Sciences, College of Veterinary Medicine, Washington State University, Pullman, WA, United States

**Keywords:** canine hemangiosarcoma, chemotherapy, multimodal therapy, prognostic factors, radiation therapy, subcutaneous and intramuscular hemangiosarcoma

## Abstract

**Introduction:**

Subcutaneous (SC) and intramuscular (IM) hemangiosarcoma (HSA) in dogs are aggressive malignancies with high rates of recurrence and metastasis. Surgery is the primary treatment, but incomplete excision is common, and the role of radiation therapy (RT) remains unclear.

**Methods:**

This single-institution retrospective study evaluated outcomes and toxicities in dogs with SC and IM HSA treated with hypofractionated RT using either 24 Gy in three 8-Gy fractions (days 0, 7, and 21) or 30 Gy in five 6-Gy fractions every other day, with or without surgery and chemotherapy.

**Results:**

Fifty-three dogs were included: 27/53 (51%) SC HSA and 26/53 (49%) IM HSA; 35/53 (66%) had macroscopic disease at RT. Overall, 47/53 (89%) received chemotherapy, of which 42/47 (89%) received doxorubicin; 35/42 (83%) received it concurrently with the first RT fraction. Among 30 evaluable dogs, the RT objective response rate (ORR) was 93% (37% complete, 56% partial). Local progression or recurrence (LPR) occurred in 11/36 (30%) dogs after a median of 128 days, predominantly with macroscopic disease (10/11). Median time to progression (TTP) was not reached (95% CI, 621 days–not reached). In an exploratory multivariable Cox analysis, metastasis was associated with increased hazard of LPR (HR, 14.95; 95% CI, 2.84–78.72; *p* = 0.0014), whereas surgery was associated with a lower hazard, but not significantly (HR, 0.15; 95% CI, 0.018–1.24; *p* = 0.079). Overall median survival time (MST) was 306 days (95% CI, 284–875). On univariable analysis, anemia, absence of surgery, macroscopic disease at RT, metastasis, and tumor size at diagnosis >6 cm were associated with shorter survival. Dogs undergoing surgery prior to RT had longer survival (MST, 2059 vs. 287 days; *p* = 0.007). In multivariable analysis, anemia remained independently associated with increased hazard of death (HR, 3.20; 95% CI, 1.38–7.45; *p* = 0.0069), whereas metastasis, surgery, and tumor size were not independently associated with survival. Chemotherapy was not associated with survival. Radiation-associated adverse events were generally mild.

**Conclusion:**

Hypofractionated RT, as part of multimodal treatment in most dogs, was associated with a high ORR and may provide durable local control in dogs with non-visceral HSA.

## Introduction

Hemangiosarcoma (HSA), a highly aggressive malignant tumor of endothelial origin, is characterized by early and widespread metastasis (1, 2). In dogs, subcutaneous (SC) and intramuscular (IM) forms represent a clinically significant subset of cases (3). Surgical excision remains the primary treatment modality; however, due to the infiltrative nature of these tumors and their high risk of local recurrence, adjuvant therapies are frequently incorporated into treatment plans (4, 5). Among these, radiation therapy (RT) is commonly considered to improve local tumor control and potentially impact survival outcomes, although its definitive role in this setting remains uncertain (4–6).

The use of RT for microscopic disease following incomplete surgical excision has been evaluated in a limited number of studies, which have been characterized by small sample sizes and inconsistent results. In one retrospective analysis, dogs with SC and IM HSA treated with surgery and doxorubicin-based chemotherapy experienced prolonged disease-free intervals (DFI) and median survival times (MST); however, a clear benefit of RT could not be demonstrated (4). Notably, dogs with SC HSA that did not receive RT had significantly longer DFI (*p* < 0.001) and a trend toward longer MST (*p* = 0.052) compared with those that received RT. Additionally, among five dogs with incompletely excised tumors treated with definitive-course RT, local control was limited, with short response durations and three recurrences (4). In contrast, Shiu et al. reported no local recurrence in three dogs with incompletely resected tumors treated with RT, highlighting the variability in reported outcomes (5).

Evidence supporting the use of RT in the gross disease setting is even more limited. Hillers et al. evaluated palliative RT in 20 dogs with non-splenic HSA using total doses ranging from 6 to 24 Gy and reported a 75% objective response rate (ORR) despite a short overall survival (95 days) in a heterogeneous population (6). Seventeen tumors involved soft tissues; however, the extent of invasion (e.g., SC or IM) could not be determined in many cases because most diagnoses were based on incisional biopsies. The remaining three tumors were located in the retroperitoneal space. Most dogs receiving 24 Gy were treated with 8 Gy × 3 fractions on days 0, 7, and 21, whereas three dogs received 6 Gy × 4 fractions on days 0, 7, 21, and 28 (6). McDonald et al. evaluated a 20-Gy palliative protocol delivered as 4 Gy × 5 consecutive daily fractions across multiple tumor types (7). The cohort included three dogs with macroscopic SC HSA, two of which demonstrated a reduction in tumor size, with response durations of 0.8 and 4.9 months; survival times for the three dogs were 1, 1.4, and 4.9 months (7).

Together, these studies provide limited but supportive evidence that hypofractionated RT can produce measurable tumor responses in dogs with macroscopic nonsplenic HSA. However, the small number of dogs with clearly characterized SC or IM HSA and the heterogeneity of the study populations, which included other nonsplenic presentations such as retroperitoneal HSA, limit conclusions regarding efficacy. Consequently, the contribution of RT specifically to outcomes in dogs with SC and IM HSA remains uncertain.

Overall, the current literature evaluating RT in dogs with SC and IM remains limited, with small cohorts, heterogeneous treatment protocols, and conflicting results, particularly with respect to its impact on local control and survival in both microscopic and macroscopic disease settings (1, 2, 4–7).

A hypofractionated protocol was selected because canine HSA is characterized by a high metastatic potential and poor long-term prognosis (1, 2), making prolonged definitive-intent radiation protocols less attractive in many patients. In addition, as a mesenchymal tumor, HSA may share radiobiologic characteristics with other sarcomas, which are generally considered to have a relatively low α/β ratio (8). Tumors with a low α/β ratio are thought to derive greater biologic benefit from larger radiation doses per fraction, as higher fraction sizes are required to achieve an equivalent biologic effect compared with conventionally fractionated protocols (8). Therefore, treatment was primarily aimed at achieving local tumor control while minimizing treatment burden, reducing the number of anesthetic events, and allowing timely administration of systemic therapy.

The objectives of this study were to describe RT objective response rate (ORR), local progression or recurrence (LPR), time to progression (TTP), median survival time (MST), prognostic factors associated with LPR and survival, and treatment-related toxicities in dogs with SC and IM HSA treated with hypofractionated RT and chemotherapy.

## Materials and methods

This single-institution retrospective study included dogs that received hypofractionated RT for SC and IM HSA. The RT database was searched for all dogs with histologically or cytologically confirmed SC or IM HSA, with microscopic or macroscopic disease, with or without metastasis at initial evaluation that underwent hypofractionated RT between 2005 and 2023.

Data recorded included signalment (breed, age, sex, weight), clinical signs, HSA locations as SC or IM, clinicopathological data, histopathologic criteria including mitotic count and mitotic score (9), clinical stage, combination with surgery or chemotherapy before or after RT, RT protocol, RT and chemotherapy toxicities, LPR, and cause of death. All medical records were reviewed and supplemented, when necessary, by contacting referring veterinarians and/or owners to obtain updated information regarding disease status, recurrence, survival, and cause of death.

Computed tomography (CT) findings were reviewed when available and were used to determine tumor location. Hemangiosarcomas were categorized as SC or IM based on CT and/or histopathology assessment.

Histopathologic reports were reviewed when available. Mitotic figures were counted within an area of 2.37 mm^2^ and recorded as a continuous variable. In addition, tumors were assigned a mitotic score according to the grading scheme described by Moore et al. (9), with scores defined as 0 (0–10 mitoses/2.37 mm^2^), 1 (11–20 mitoses/2.37 mm^2^), 2 (21–30 mitoses/2.37 mm^2^), and 3 (>30 mitoses/2.37 mm^2^). These categories were used for subsequent statistical analyses.

Radiation therapy was delivered with a linear accelerator (Elekta SLi 15, Philips Medical Systems N.A., Bothell, WA, United States) using 6 MV photons or 8–12 MeV electron beams using a single field. All treatments were planned by the same board-certified radiation oncologist (JF) using manual or three-dimensional conformal RT (3D-CRT) radiation planning.

For manually calculated electron plans, treatment volume was estimated using a simple cuboidal model (length × width × height) to guide field design and dose distribution. In cases without a measurable gross tumor volume, treatment planning was based on the surgical scar and the estimated extent of microscopic disease. The extent of microscopic disease and treatment margins were determined by the treating radiation oncologist based on the expected biological behavior and infiltrative nature of sarcomas, available histopathologic information, tumor location, and anatomic constraints. Treatment margins were applied laterally and in depth, with adjustments based on anatomic constraints and adjacent OARs. Treatment field margins were usually set at 3 cm from the tumor or surgical scar.

Field-shaping blocks (square and rectangular) were used for specific sizes in electron beam planning. Electron energy was selected based on lesion depth and proximity to organs at risk (OARs). Dose was prescribed to an appropriate isodose line according to electron depth dose tables generated for the linear accelerator by a medical physicist, to ensure target coverage. The prescribed isodose line ranged from 90% to 100% in this study. The planning goal was to cover at least 95% of the target volume with the prescribed dose.

For CT-based planning and treatment, patients were anesthetized and positioned in lateral or sternal recumbency (depending on the affected area) with stabilization provided by an individually moldable vacuum positioning mattress. Multiple vacuum positioning mattresses were used over the retrospective study period, and the specific manufacturer and model used for individual patients were not consistently documented. Dogs with tumors involving the head and neck were additionally positioned using a custom-made thermoplastic bite block fabricated in-house.

Pre- and post-contrast CT image sets were imported into the Prowess Panther Treatment Planning System version 5.01–5.1 (Prowess, Inc. 1844 Clayton Road, Concord, CA, 94520), depending on the year of treatment. Treatments were 3D-CRT plans. The gross tumor volume (GTV) was contoured to delineate visible macroscopic tumor on contrast-enhanced CT images. For macroscopic disease, the clinical target volume (CTV) encompassed the GTV plus a non-uniform margin for potential microscopic extension into surrounding tissues; margins were generally up to 3 cm in soft tissue such as muscle, but reduced when constrained by the skin surface, adjacent anatomy, or OAR. For postoperative microscopic disease, the CTV was based on the surgical scar/tumor bed and the estimated extent of microscopic disease, with margins similarly modified according to anatomic constraints. Regional lymph nodes were included only when clinically indicated. A uniform three-dimensional margin of 3 mm was added to the CTV to create the planning target volume (PTV), accounting for internal motion, patient movement, and setup uncertainty. Planning objectives included 99% of the GTV (D99) and 95% of the PTV (D95) receiving 100% of the prescribed total dose.

Kilovoltage port films were used at each treatment to ensure accurate tumor positioning. Digital films (Fujifilm type IP cassette, Fuji Photofilm Co. LTD, Tokyo, Japan) were used for larger fields and improved tissue discrimination than onboard imaging and were reviewed by one author (JF).

Two hypofractionated radiation protocols were employed: a total prescribed dose of 24 Gy delivered in three 8-Gy fractions on days 0, 7, and 21, or a total prescribed dose of 30 Gy delivered in five 6-Gy fractions administered every other day. Selection of the protocol was based on the feasibility of coordinating doxorubicin administration with RT treatments (most practical with the 8 Gy × 3 fraction protocol, in which doxorubicin was administered on days 0 and 21), logistical considerations including owner ability to travel for multiple outpatient visits versus short-term hospitalization for the every-other-day 6 Gy × 5 fraction protocol, and financial factors, as the three-fraction protocol was more cost-effective and required fewer anesthetic events. Although the fractionation schedules differed, the protocols were selected to provide a comparable biologically effective dose (BED), thereby maintaining a broadly similar modeled biological effect while allowing flexibility in treatment planning and patient management. BED calculations based on the linear-quadratic model do not account for the different overall treatment times of these protocols (days 0, 7, and 21 for 8 Gy × 3 versus approximately 9 days for 6 Gy × 5); therefore, BED comparison alone may not fully capture differences in tumor repopulation or normal-tissue effects between schedules.

Local progression was defined as an increase in size of a treated macroscopic tumor following an initial response to therapy, whereas recurrence was defined as the reappearance of a previously surgically removed tumor after a documented period of no evidence of disease following adjuvant RT. For subsequent analyses, local progression and local recurrence were combined into a single outcome, herein referred to as local progression or recurrence (LPR), rather than evaluated independently. Tumor size was categorized as ≤ 4 cm, 4–6 cm, and > 6 cm for statistical analysis, consistent with the categories used by Shiu et al. (5).

Tumor response to RT was assessed retrospectively according to the Veterinary Cooperative Oncology Group Response Evaluation Criteria for Solid Tumors in Dogs (VCOG RECIST v1.0) (10). Response was classified as complete response (CR) defined as disappearance of all target lesions; partial response (PR) defined as ≥ 30% decrease in the sum of the diameters of target lesions; progressive disease (PD) defined as ≥ 20% increase in the sum of diameters of target lesions, or the appearance of new lesions; and stable disease (SD) defined as <30% reduction or <20% increase in the sum of diameters of target lesions (10).

Tumor response and LPR were assessed retrospectively from available medical records. When recorded, measurable superficial lesions were assessed by physical examination with caliper measurements; cross-sectional imaging was reviewed when it had been performed for clinical reassessment. Systemic progression was assessed using the restaging studies available for each dog, which could include thoracic radiographs or thoracic CT, abdominal ultrasonography, and other clinically indicated imaging. Response and progression assessments were based on the best available documentation for each patient. The institutional recommendation was for patients to undergo a post-treatment recheck approximately 3–4 weeks after completion of RT to assess resolution of acute treatment-related adverse effects and evaluate the treated tumor site. This initial recheck could be performed at the referring veterinarian (rDVM). Thereafter, surveillance was recommended every 3–4 months and generally included a physical examination to assess for LPR, thoracic radiographs, possible abdominal ultrasound, and imaging of the treated tumor site when clinically indicated. Follow-up could be performed either at our institution or by the rDVM, depending on logistical considerations and owner preference.

Radiation therapy-associated adverse events were retrospectively graded according to the Veterinary Radiation Therapy Oncology Group (VRTOG) v2.0 criteria (11). Acute adverse events were defined as those occurring during or within 3 months after completion of RT, whereas late adverse events were defined as those arising or persisting more than 3 months after completion of RT (11). When documentation in the medical record was insufficient to reliably determine the grade of an adverse event, the event was recorded but considered ungradable.

Chemotherapy-related toxicities were assessed retrospectively according to the Veterinary Cooperative Oncology Group Common Terminology Criteria for Adverse Events (VCOG-CTCAE v2) (12).

Median TTP was defined as the time from the start of RT to LPR. Survival time was defined as the time from diagnosis to date of death. Dogs were censored from TTP analysis if they were lost to follow up with no evidence of tumor recurrence at the last known date of contact or if they died of other known causes. If the cause of a dog’s death was uncertain and the dog had evidence of HSA at the time of death, death was attributed to HSA and it was included in all analyses. Additional therapies were allowed after disease progression.

Statistical analyses were conducted using statistical software (R Studio version 4.4.1). Descriptive analysis was performed using Microsoft Excel (version 16.86). Associations between categorical variables were assessed using Fisher’s exact tests. The MST and TTP data were plotted using Kaplan–Meier curves and comparisons between group variables were analyzed with the log-rank test with significance set at *p* < 0.05. Prognostic factors were further evaluated using Cox proportional hazards regression. Univariable Cox models were reported for TTP. Given the limited number of LPR events, a parsimonious exploratory multivariable Cox proportional hazards model was performed for time to LPR using clinically relevant variables identified from the univariable analyses. Because of the limited number of events, results of this model were considered exploratory and interpreted cautiously. For overall survival, clinically relevant variables based on previous studies and biologic plausibility were included in a multivariable Cox proportional hazards model; hazard ratios (HRs), 95% confidence intervals (CIs), and *p* values were reported.

## Results

Fifty-three dogs with cytologically or histopathologically confirmed SC and IM HSA were included in the study.

### Signalment

The median age was 10 years (range, 4–14 years) and the median weight was 28.5 kg (range 7.3–76.6 kg). There were 24 spayed females and 29 males (28 neutered). The most common pure breeds were Labrador Retriever (8), Golden Retriever (4), Miniature Schnauzer (3), German Shepherd (2), Whippet (2), Australian Shepherd (2), Border Collie (2), Cairn Terrier (2) and 1 each of 14 additional pure-breed dogs; 14 dogs were mixed breed.

### Clinical signs, HSA location and stage

Hemangiosarcomas were categorized as SC or IM based upon CT imaging and histopathology when available. Twenty-seven dogs (51%) were diagnosed with SC HSA and 26 dogs (49%) with IM HSA. All dogs had clinical signs, including mass effect (50), pain (9), lameness (8), bleeding/ulceration (5), swelling (3), lethargy (3), drooling/dysphagia (2), and dyspnea (1).

Tumor anatomic sites included pelvic limb (13/53, 24%), head (11/53 20%), inguinal region (8/53, 15%), thoracic limb (7/53, 13%), epaxial musculature (4/53, 8%), neck (4/53, 8%), axilla (3/53, 6%), abdominal wall/flank (2/53, 4%), and perineal region (1/53, 2%).

Staging tests performed were variable between dogs. Complete blood count and serum biochemistry panel results were available at diagnosis for all 53 dogs. Anemia was identified in 11 dogs (21%) and thrombocytopenia in 10 dogs (19%), based on the reference intervals of the reporting laboratories. No consistent abnormalities were noted on serum biochemical analysis.

Fifty-two of 53 dogs (98%) had thoracic radiographs or computed tomography and abdominal ultrasound performed at or around the time of diagnosis; pulmonary nodules consistent with metastatic disease were identified in 14 dogs (27%) and intraabdominal metastasis confirmed via cytology in 4 dogs (8%). Forty-six dogs (87%) had echocardiogram and 2 dogs (5%) had right auricle masses. Staging in one dog consisted only of a complete blood count and serum biochemistry panel, with no diagnostic imaging performed. The overall known metastatic rate at the time of diagnosis was 38% (20/52).

### Tumor identification and treatments

Five dogs (9%) had a cytological diagnosis, and 48 dogs (91%) had a histopathological diagnosis. All cytologic evaluations were performed by board-certified clinical pathologists and were consistent with sarcoma with features suggestive of HSA. Mitotic count was available in 37 cases, with a median of 11 mitotic figures per 2.37 mm^2^ (range 0–80). Dogs with microscopic disease at RT time had a lower median mitotic count (7; range, 1–40) compared with those with macroscopic disease (12; range, 0–80). Based on mitotic figures per 2.37 mm^2^, mitotic scores were: 0 (0–10; *n* = 18), 1 (11–20; *n* = 7), 2 (21–30; *n* = 7), and 3 (>30; *n* = 5).

Tumor size at diagnosis was available for 50 dogs: 16 (≤4 cm), 10 (>4 cm and ≤6 cm), and 24 (>6 cm) (Table 1). At RT time, 35 (66%) had macroscopic disease, 4 (≤4 cm), 7 (>4 cm and ≤6 cm), and 21(>6 cm); median tumor size was >6 cm. Three cases lacked measurable size (multiple masses, no measurement, or non-measurable facial swelling). Eighteen dogs (34%) had surgery prior to RT; margins were dirty (9/18, 50%), narrow (7/18, 38%), complete (1/18, 6%), and unavailable (1/18, 6%), with a median tumor size <4 cm.

**Table 1:** Factors associated with survival in dogs with SC and IM HSA treated with hypofractionated RT.

Variable for survival	Category	No. of dogs	MST (days)	95% CI	*p*-value
HSA locations	IM	26	301	256–960	0.47
	SC	27	534	280-NR	
Tumor size at diagnosis	≤4 cm	16	2059	395-NR	**0.00044**
	>4 cm and ≤ 6 cm	10	638	280-NR	
	> 6 cm	24	284	199–339	
Tumor size at RT	≤4 cm	4	939	145-NR	0.21
	>4 cm and ≤ 6 cm	7	301	256-NR	
	> 6 cm	21	284	199-NR	
Thrombocytopenia	No	43	395	289–1729	0.053
	Yes	10	256	165-NR	
Anemia	No	42	647	302–2059	**<0.0001**
	Yes	11	222	135-NR	
Mitotic score	Score 0	18	2059	278-NR	0.2
	Score 1	7	280	145-NR	
	Score 2	7	534	256-NR	
	Score 3	5	156	147-NR	
Metastasis	No	33	742	301-NR	**0.0037**
	Yes	19	280	177-NR	
Surgery	No	35	287	224–647	**0.0074**
	Yes	18	2059	395-NR	
RT protocol	8Gy × 3 fractions	44	302	280–875	0.57
	6Gy × 5 fractions	9	339	280-NR	
Response to RT	CR	11	263	149-NR	0.38
	PR	17	280	256–742	
	SD	2	964	199-NR	
Chemotherapy	No	6	2,406	395-NR	0.092
	Yes	47	301	280–785	
Recurrence	No	25	742	280-NR	0.2
	Yes	11	287	199-NR	

CI, confidence interval; NR, not reached; RT, radiation therapy; SC, subcutaneous; IM, intramuscular. Bold values indicate statistical significance (*p* < 0.05).

### Radiation therapy

Of the 53 dogs, 24 (45%) had manual planning and were treated with a single electron beam field (8–12 MeV) and 29 (55%) had 3D-CRT delivered via 6 MV photons with either a single beam or parallel opposed beams. The regional lymph node was only irradiated in one patient. Regarding RT protocols, 83% (44/53) received 8 Gy × 3 fractions (total dose 24 Gy), and 17% (9/53) received 6 Gy × 5 fractions (total dose of 30 Gy). Three dogs underwent re-irradiation at 91, 489, and 706 days following the initial treatment; the first was treated with electrons and the latter two with photons.

### Radiation therapy-related toxicosis

Thirty-two adverse events were documented in 23 dogs. Acute adverse events included moist dermatitis (*n* = 6), uveitis with keratoconjunctivitis sicca (*n* = 1), and tumor ulceration (*n* = 1); these events could not be reliably graded because the available medical records contained insufficient information to assign a VRTOG v2.0 grade. Late adverse events included grade 1 alopecia (*n* = 15), grade 1 hyperpigmentation (*n* = 5), grade 1 leukotrichia (*n* = 3), and grade 4 phthisis bulbi (*n* = 1). The dog with phthisis bulbi did not have a documented adverse impact on quality of life. Twenty dogs had no documented RT-associated adverse events, and toxicity information was unavailable for 10 dogs.

### Chemotherapy

Forty-seven dogs (89%) received chemotherapy, of which 42 (89%) received doxorubicin with a median of 3 doses (1–6 doses). The starting doxorubicin dosage was 30 mg/m^2^ (*n* = 33) or 1 mg/kg if <15 kg (*n* = 5); 3 dogs received lower doses (15–25 mg/m^2^), and dosage was unrecorded in 1. Dose adjustments were at clinician discretion. Thirty-five dogs (83%) received doxorubicin on the same day as the first RT dose, 4 (10%) before RT, and 1 (2%) after RT completion; timing was not recorded in 2 (5%). Five of 47 dogs (10%) received metronomic lomustine (2.87 mg/m^2^ orally once daily): 1 before RT, 2 after RT completion, and 2 with unknown timing.

After completion of the RT and doxorubicin, 15 dogs received additional chemotherapy. Thirteen received metronomic chemotherapy, including lomustine (7), cyclophosphamide (4), and chlorambucil (2). One dog received a single lomustine dose (54 mg/m^2^), and one received toceranib (unknown dosage).

### Chemotherapy-related toxicosis

Episodes of toxicosis were recorded 50 times among 31 dogs receiving chemotherapy, with 30 of those dogs experiencing events associated with doxorubicin (Table 2). No adverse effects were reported in 9 dogs, and toxicity data were unavailable for 7 dogs. Eight dogs required doxorubicin dose reductions due to toxicosis: grade 1 neutropenia (1), grade 1 neutropenia and gastrointestinal side effects (1), grade 3 neutropenia (1), grade 4 neutropenia (3), and neutropenia of unspecified grade (2). The only toxicity observed with metronomic lomustine was hepatotoxicity in one dog; data were unavailable for the remaining four.

**Table 2:** Reported episodes of toxicosis following doxorubicin therapy.

Toxicosis	Grade 1	Grade 2	Grade 3	Grade 4	Grade not recorded
Neutropenia	8	1	5	3 (1 febrile neutropenia)	3
Thrombocytopenia	4	3	-	-	1
GI – Vomiting, diarrhea, and/or anorexia	-	-	-	-	12
Lethargy	-	-	-	-	7
Alopecia	-	-	-	-	1
Skin hyperpigmentation	-	-	-	-	1
VPC’s	-	-	-	-	1

### Outcome

Five dogs were lost to follow-up, and three were still alive at the time of data compilation. Median follow-up time was 1,346 days (range, 134–2,136 days).

Of the 35 dogs with macroscopic disease that underwent RT, 30 had response data; 28 (93%) achieved an objective response, including 11 (37%) CR and 17 (56%) PR.

Among these 30 dogs, 22 received doxorubicin concurrently with RT; 21/22 achieved an objective response (10 CR and 11 PR), while one had SD. Four dogs had received doxorubicin before RT and had either failed to respond or were no longer responding to doxorubicin when RT was initiated; all four subsequently achieved an objective response following RT (three PR and one CR). One additional dog had a PR documented at the time metronomic lomustine was initiated one week after completion of RT, and another dog had a PR documented at the time doxorubicin was initiated after completion of RT. In two dogs, the timing of chemotherapy relative to RT could not be reliably determined from the medical records. Dates of initial and maximal tumor response were not consistently documented; therefore, time to response and the association between concurrent doxorubicin administration and response timing could not be reliably evaluated.

Local progression or recurrence data were available for 36/53 dogs, of which 11 (30%) experienced LPR after a median of 128 days (range, 44–1,675 days); median TTP was not reached (95% CI: 621 days–not reached). Most events occurred in dogs with macroscopic disease at RT time (10/11), including 5 PR, 3 CR, and 2 SD (median mitotic count was 24; mitotic score 2). Out of the animals with microscopic disease, only one recurrence occurred in a dog with microscopic disease following surgery (<4 cm SC HSA on the thoracic limb, dirty margins, mitotic count 1; mitotic score 0), 317 days after RT. Among dogs categorized by best response, median TTP was not reached for dogs achieving CR (95% CI, 180 days–NR), 397 days for dogs achieving PR (95% CI, 114 days–NR), and 906 days for dogs with SD (95% CI, 138 days–NR); response category was not significantly associated with LPR (*p* = 0.43).

Margin status was available in 17/18 dogs undergoing surgery prior to RT. Among 12 with known recurrence status, no LPR occurred with clean (*n* = 1) or narrow margins (*n* = 5), while 1/6 (17%) with dirty margins developed LPR.

Use of chemotherapy was not associated with LPR (*p* = 0.29). Among six dogs without chemotherapy, five had prior surgery (margins: two narrow, two dirty) with no recurrences, while one dog with gross disease (SD) developed LPR at 138 days post-RT.

Among dogs with evaluable LPR data, several clinicopathologic variables were significantly associated with TTP (Table 3). Surgery prior to RT significantly improved local control (*p* = 0.0079), with median TTP not reached versus 621 days (95% CI: 173–NR) with RT alone. Tumor size at diagnosis was strongly prognostic (*p* = 0.0038), with tumors >6 cm having shorter TTP (173 days, 95% CI: 119–NR), versus not reached for tumors ≤6 cm. Similarly, anemia at diagnosis was associated with significantly shorter TTP (median 173 days, 95% CI: 119–NR vs. not reached; *p* = 0.0023). Higher mitotic score was also associated with worse local control (*p* = 0.028), with score 3 tumors experiencing rapid progression (median 56 days, 95% CI: 44–NR), while lower scores had prolonged disease control. Metastasis at diagnosis was a strong negative prognostic factor (*p* < 0.0001), with a median TTP 119 days (95% CI: 114–NR) compared to not reached in dogs without metastasis.

**Table 3:** Factors associated with local progression or recurrence (LPR) in dogs with SC and IM HSA treated with hypofractionated RT.

Variable for LPR	Category	No. of dogs	Median TTP (days)	95% CI	*p*-value
HSA locations	IM	15	NR	621-NR	0.9
	SC	21	1,675	317-NR	
Tumor size at diagnosis	≤4 cm	13	NR	1,675-NR	**0.0038**
	>4 cm and ≤ 6 cm	6	NR	621-NR	
	>6 cm	15	173	119-NR	
Tumor size at RT	≤4 cm	4	1,675	NR-NR	0.072
	>4 cm and ≤ 6 cm	4	621	56-NR	
	>6 cm	13	173	119-NR	
Thrombocytopenia	No	31	NR	621-NR	0.29
	Yes	5	NR	119-NR	
Anemia	No	30	NR	1,675-NR	**0.0023**
	Yes	6	173	119-NR	
Mitotic score	Score 0	11	NR	317-NR	**0.028**
	Score 1	5	NR	NR-NR	
	Score 2	5	NR	NR-NR	
	Score 3	3	56	44-NR	
Metastasis	No	27	NR	1,675-NR	**<0.0001**
	Yes	9	119	114-NR	
Surgery	No	23	621	173-NR	**0.0079**
	Yes	13	NR	NR-NR	
RT protocol	8Gy × 3 fractions	28	NR	1,675-NR	0.28
	6Gy × 5 fractions	8	621	173-NR	
Response to RT	CR	10	NR	180-NR	0.43
	PR	11	397	114-NR	
	SD	2	906	138-NR	
Chemotherapy	No	5	NR	NR-NR	0.29
	Yes	31	1,675	317-NR	

CI, confidence interval; NR, not reached; RT, radiation therapy; SC, subcutaneous; IM, intramuscular. Bold values indicate statistical significance (*p* < 0.05).

In contrast, HSA location was not significantly associated with LPR (*p* = 0.90), with a median TTP of not reached (NR; 95% CI, 621 days–NR) for IM HSA and 1,675 days (95% CI, 317 days–NR) for SC HSA. Similarly, thrombocytopenia was not associated with LPR (*p* = 0.29), with a median TTP of NR (95% CI, 621 days–NR) in dogs without thrombocytopenia and NR (95% CI, 119 days–NR) in dogs with thrombocytopenia. No significant difference was observed between RT protocols (*p* = 0.28); dogs treated with 8 Gy × 3 fractions had a median TTP of NR (95% CI, 1,675 days–NR), whereas dogs treated with 6 Gy × 5 fractions had a median TTP of 621 days (95% CI, 173 days–NR). Likewise, response to RT was not significantly associated with LPR (*p* = 0.43), with median TTPs of NR (95% CI, 180 days–NR), 397 days (95% CI, 114 days–NR), and 906 days (95% CI, 138 days–NR) for dogs achieving CR, PR, and SD, respectively.

In univariable Cox proportional hazards analysis for time to LPR, tumor size > 6 cm (HR, 15.69; 95% CI, 1.10–223.8; *p* = 0.042) and the presence of metastatic disease at diagnosis (HR, 28.1; 95% CI, 2.99–264.24; *p* = 0.0035) were significantly associated with an increased risk of LPR. However, the effect of metastasis was associated with model instability, likely due to separation, and should be interpreted with caution. Surgery prior to RT (HR, 0.15; 95% CI, 0.02–1.25; *p* = 0.079), gross disease at the time of RT (HR, 6.83; 95% CI, 0.79–59.2; *p* = 0.079), and increasing mitotic score (HR, 1.74; 95% CI, 0.92–3.32; *p* = 0.091) demonstrated trends toward association with LPR but did not reach statistical significance. Although anemia was associated with shorter TTP in Kaplan–Meier analysis, it was not significantly associated with LPR in univariable Cox regression (HR, 5.43; 95% CI, 0.55–53.11; *p* = 0.146). Thrombocytopenia and tumor location were not significantly associated with TTP.

In an exploratory multivariable Cox proportional hazards analysis for time to LPR (Table 4), the presence of metastasis was associated with an increased hazard of LPR (HR, 14.95; 95% CI, 2.84–78.72; *p* = 0.0014), whereas surgery was associated with a lower hazard, although this did not reach statistical significance (HR, 0.15; 95% CI, 0.018–1.24; *p* = 0.079).

**Table 4:** Exploratory multivariable Cox proportional hazards analysis of factors associated with time to local progression or recurrence (LPR).

Variable	Comparison	HR	95% CI	*p*
Metastasis	Yes vs. No	14.95	2.84–78.72	**0.0014**
Surgery	Yes vs. No	0.15	0.018–1.24	0.079

HR, hazard ratio; CI, confidence interval; RT, radiation therapy. Bold values indicate statistical significance (*p* < 0.05). The analysis was exploratory because of the limited number of LPR events; estimates should therefore be interpreted cautiously.

The MST for all dogs was 306 days (95% CI, 284–875 days; Figure 1). Several variables were associated with survival (Table 1). Tumor size at diagnosis was strongly prognostic (*p* = 0.00044; Figure 2a), with MST 2059 days (95% CI, 395–NR) for tumors ≤4 cm versus 284 days (95% CI, 199–339) for >6 cm.

**Figure 1:**
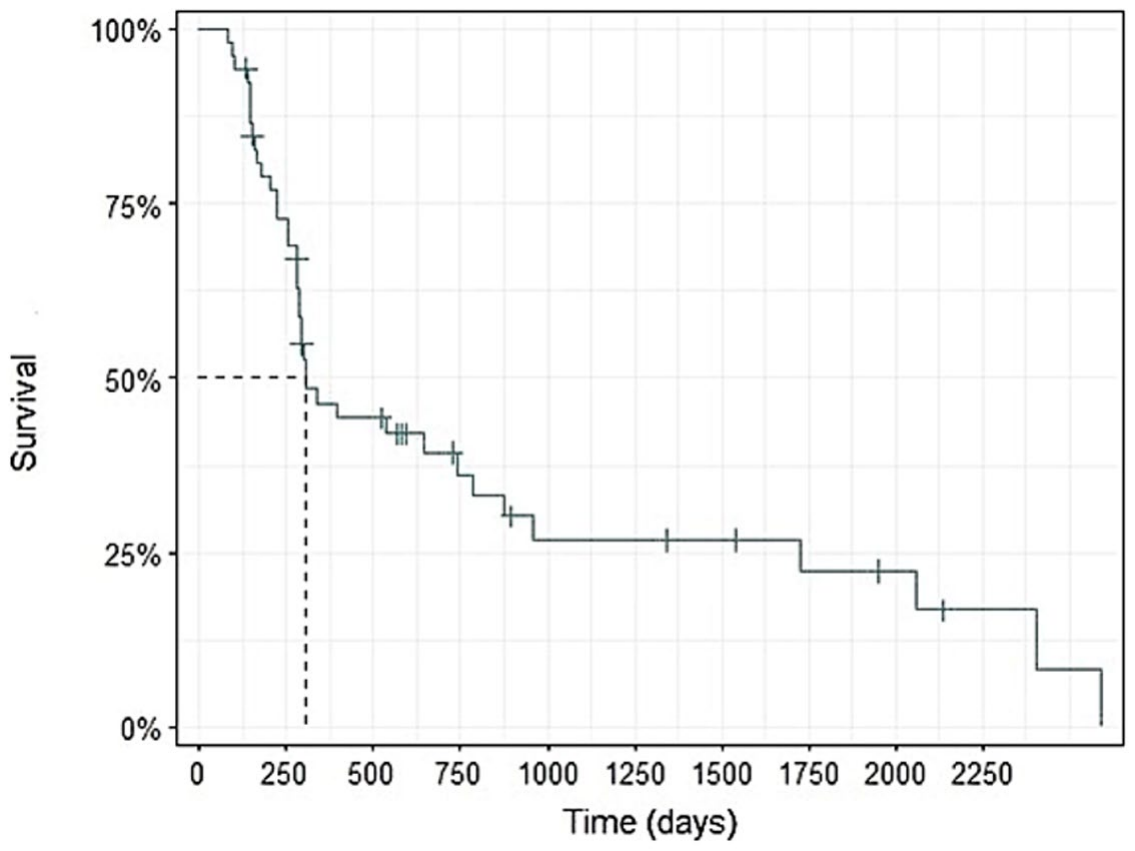
Kaplan–Meier curve showing overall survival probability over time (days) for 53 dogs with subcutaneous (SC) or intramuscular (IM) hemangiosarcoma (HSA) treated with hypofractionated radiation therapy (RT). Median survival time (MST) was 306 days (95% CI, 284–875). Tick marks indicate censored observations.

**Figure 2:**
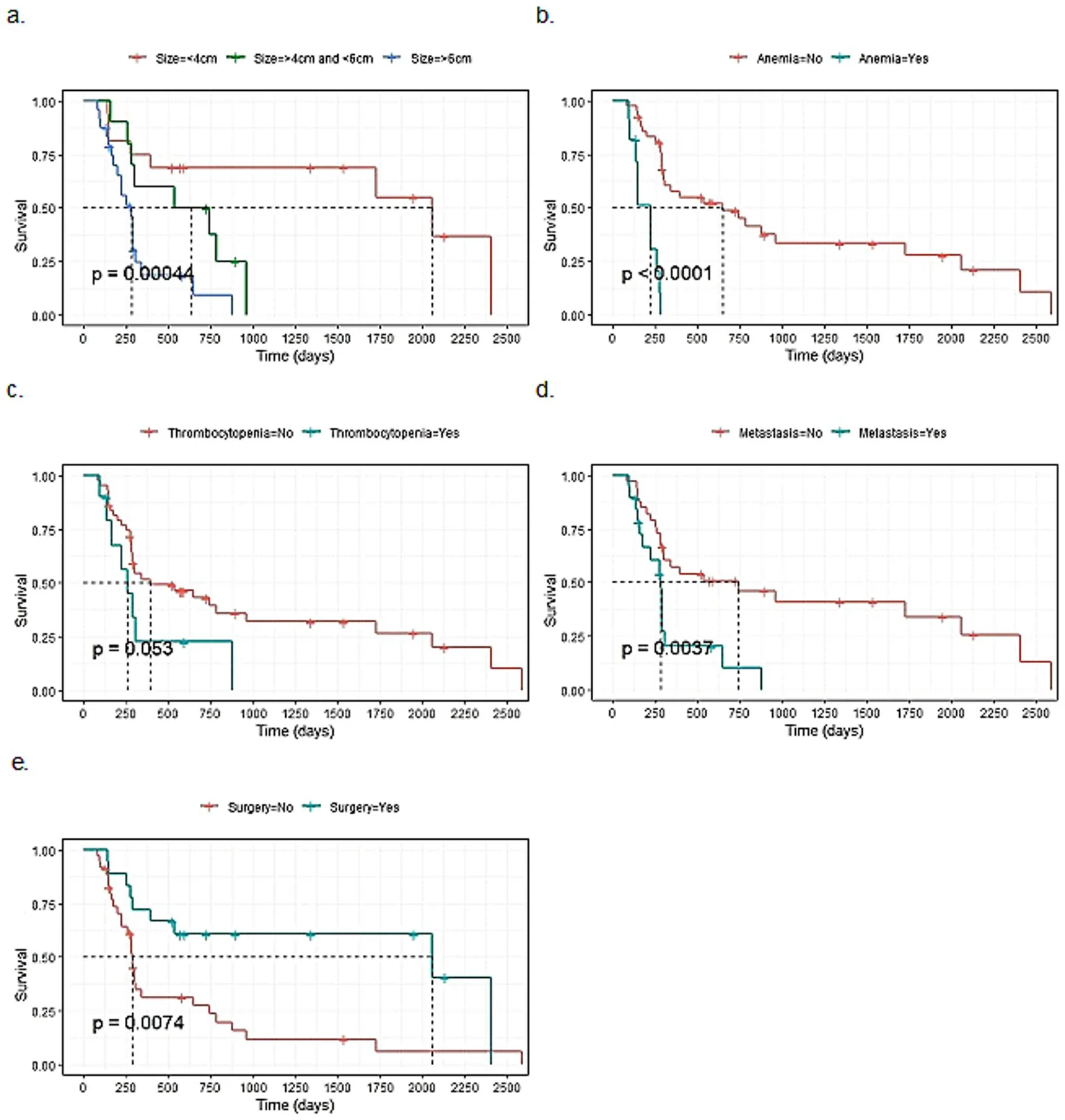
Kaplan–Meier overall survival curves according to selected variables. **(a)** Tumor size at diagnosis: ≤4 cm (*n* = 16; MST, 2,059 days), >4 to ≤6 cm (*n* = 10; MST, 638 days), and >6 cm (*n* = 24; MST, 284 days; *p* = 0.00044). **(b)** Anemia status at diagnosis: no anemia (*n* = 42; MST, 647 days) versus anemia (*n* = 11; MST, 222 days; *p* < 0.0001). **(c)** Thrombocytopenia status at diagnosis: no thrombocytopenia (*n* = 43; MST, 395 days) versus thrombocytopenia (*n* = 10; MST, 256 days; *p* = 0.053). **(d)** Metastatic status at diagnosis: no metastasis (*n* = 33; MST, 742 days) versus metastasis (*n* = 19; MST, 280 days; *p* = 0.0037). **(e)** Cytoreductive surgery prior to RT: no surgery (*n* = 35; MST, 287 days) versus surgery (*n* = 18; MST, 2,059 days; *p* = 0.0074). Tick marks indicate censored observations.

Anemia at diagnosis was associated with worse survival (*p* < 0.0001; Figure 2b), with MST 222 days (95% CI: 135–NR) versus 647 days (95% CI: 302-2059) in non-anemic dogs. Thrombocytopenia showed a negative trend (*p* = 0.053; Figure 2c). These abnormalities were more common in dogs with metastatic disease: anemia was present in 64% of dogs with metastasis versus 29% without, and thrombocytopenia in 32% versus 12%, respectively. Metastasis at diagnosis was associated with shorter survival (280 days, 95% CI: 177–NR vs. 742 days, 95% CI: 301–NR; *p* = 0.0037; Figure 2d).

Surgery prior to RT improved survival (*p* = 0.0074; Figure 2e), with MST 2059 days (95% CI: 395–NR) versus 287 days (95% CI: 224–647).

Conversely, HSA location (*p* = 0.47), tumor size at RT (*p* = 0.21), mitotic score (*p* = 0.2), RT protocol (*p* = 0.57), response to RT (*p* = 0.38), chemotherapy (*p* = 0.092), and LPR (*p* = 0.2) were not significantly associated with survival (see Figure 3).

**Figure 3:**
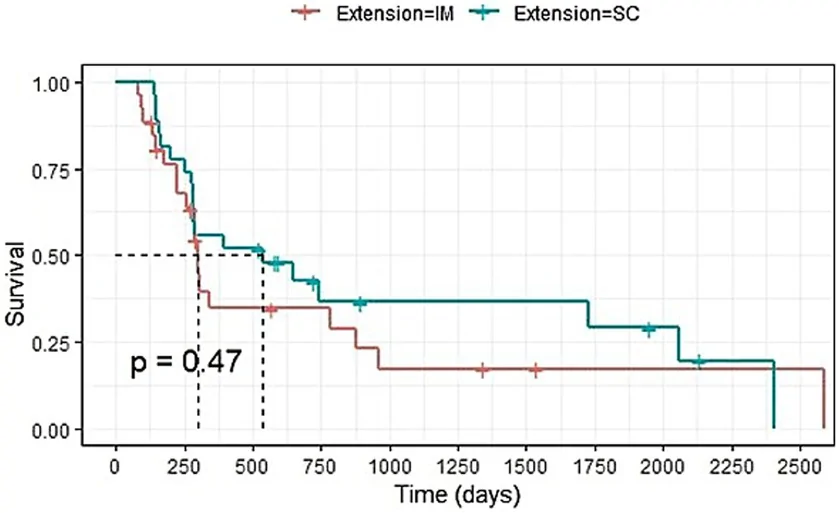
Kaplan–Meier overall survival curves comparing 26 dogs with intramuscular (IM) hemangiosarcoma (HSA) and 27 dogs with subcutaneous (SC) HSA. Median survival time (MST) was 301 days for dogs with IM HSA and 534 days for dogs with SC HSA (*p* = 0.47). Tick marks indicate censored observations.

Univariable Cox regression analyses were initially performed to evaluate potential prognostic factors associated with overall survival. Anemia was significantly associated with decreased survival (HR, 8.36; 95% CI, 2.85–24.55; *p* = 0.0001), surgery prior to RT was associated with improved survival (HR, 0.36; 95% CI, 0.14–0.93; *p* = 0.035), whereas gross disease at the time of RT was associated with decreased survival (HR, 2.75; 95% CI, 1.08–6.99; *p* = 0.035). Dogs with tumors > 6 cm had a significantly greater risk of death compared with dogs with tumors ≤ 4 cm (HR, 4.48; 95% CI, 1.41–14.18; *p* = 0.011). Metastasis was significantly associated with overall survival when evaluated as a global variable (likelihood ratio test, *p* = 0.046), although individual categories were not statistically significant. Thrombocytopenia and mitotic score showed trends toward an association with overall survival but did not reach statistical significance. Tumor location (SC vs. IM) was not significantly associated with survival.

Variables considered clinically relevant based on previous studies and biologic plausibility were subsequently included in a multivariable Cox proportional hazards regression analysis for survival (Table 5). The final model included 37 dogs, with 24 events recorded. Anemia at the time of diagnosis remained independently associated with decreased overall survival (HR, 3.20; 95% CI, 1.38–7.45; *p* = 0.0069). Metastasis (HR, 1.81; 95% CI, 0.70–4.66; *p* = 0.220) and surgery (HR, 0.58; 95% CI, 0.20–1.69; *p* = 0.319) were not independently associated with overall survival. Compared with tumors ≤4 cm, tumors >4–6 cm (HR, 2.63; 95% CI, 0.75–9.23; *p* = 0.131) and >6 cm (HR, 3.29; 95% CI, 0.81–13.43; *p* = 0.097) had higher estimated hazards of death, although neither association reached statistical significance. Gross disease, thrombocytopenia, tumor extension, and mitotic score were not independently associated with survival after adjustment for the other variables.

**Table 5:** Multivariable Cox proportional hazards analysis for overall survival.

Variable	Comparison	HR	95% CI	*p*
Anemia	Yes vs. No	3.20	1.38–7.45	**0.0069**
Metastasis	Yes vs. No	1.81	0.70–4.66	0.220
Surgery	Yes vs. No	0.58	0.20–1.69	0.319
Tumor size at diagnosis	>4–6 cm vs. ≤ 4 cm	2.63	0.75–9.23	0.131
Tumor size at diagnosis	>6 cm vs. ≤ 4 cm	3.29	0.81–13.43	0.097

The model included 37 dogs and 24 events. HR, hazard ratio; CI, confidence interval; RT, radiation therapy; SC, subcutaneous; IM, intramuscular. Bold values indicate statistical significance (*p* < 0.05).

Cause of death was available for 35 dogs. Eight dogs (23%) were euthanized for causes unrelated to HSA, including poor mobility (2 dogs), age-related causes of unspecified reason (2 dogs), trauma, acute leukemia, primary pulmonary carcinoma (lungs were not in the RT field; no evidence of HSA at necropsy), and laryngeal paralysis (pre-existing chronic condition; potential contribution of anesthesia cannot be excluded, as death occurred 8 days following the final RT treatment in a reirradiation case; the radiation field did not include the head or neck). The remaining 27 dogs (77%) were euthanized due to disease progression, unknown causes, or were found deceased and were therefore classified as tumor-related deaths.

## Discussion

To the authors’ knowledge, this study represents the first and largest cohort dedicated exclusively to dogs with SC and IM HSA treated with RT, avoiding the heterogeneity introduced by inclusion of other nonsplenic HSA subtypes reported in previous studies. The results demonstrate that hypofractionated RT, delivered in conjunction with chemotherapy in most dogs, provides clinically meaningful disease control. The ORR of 93% observed in dogs with measurable disease is comparable to the approximately 75% ORR reported by Hillers et al. (6) and McDonald et al. (7) in dogs with nonsplenic HSA treated with a similar hypofractionated radiation protocol. In addition, complete responses were observed in 37% of dogs in the present study, compared with 20% in the Hillers cohort. These findings support the reproducibility of hypofractionated RT as an effective local treatment strategy for gross HSA. While direct comparison between studies should be interpreted cautiously because Hillers et al. included a more heterogeneous population of nonsplenic HSA, including retroperitoneal tumors, the consistency of response rates across studies suggests meaningful response of this tumor type. Importantly, dogs with macroscopic disease at the time of RT achieving complete or partial responses frequently maintained local tumor control for prolonged periods, with a median TTP of 397 days among dogs achieving a PR and median TTP not reached among dogs achieving a CR. Although response category was not significantly associated with TTP in the present study, these findings suggest that hypofractionated RT can provide durable local disease control in a subset of patients. Nevertheless, LPR occurred in 30% of evaluable dogs, indicating that despite excellent initial response rates, durable local control remains a therapeutic challenge.

The present findings also provide insight into the potential role of RT for microscopic disease following surgery. Of the 11 LPR events observed, 10 occurred in dogs treated for gross disease, whereas only one event was documented in a dog treated with surgery followed by adjuvant RT for microscopic disease. These findings suggest that hypofractionated RT may provide adequate local control of microscopic residual disease. The use of RT for microscopic residual disease following incomplete surgical excision has been investigated in a limited number of studies, with conflicting results regarding its impact on local recurrence and survival. In the study by Bulakowski et al., five dogs received definitive-intent RT after incomplete excision; however, RT was not associated with improved disease-free interval or survival, and three of the four evaluable dogs developed local recurrence (4). Comparison with the present study is challenging because substantially different radiation protocols were employed. Whereas Bulakowski et al. primarily used conventionally fractionated definitive-intent RT protocols, all dogs in the present study received hypofractionated RT, which may produce different radiobiologic effects in sarcomas. Although direct comparison is limited by the small number of irradiated dogs in the Bulakowski study, the low recurrence rate observed in the microscopic disease subgroup of the present study supports the potential efficacy of hypofractionated RT for control of microscopic residual disease. Shiu et al. reported a local recurrence rate of 16% (10/61 dogs) following treatment of SC and IM HSA; however, the authors did not specify whether the dogs that developed recurrence had received RT, making it impossible to determine the contribution of RT to local control (5). Taken together, the present study suggests that RT may have a role in managing microscopic disease following surgery, although larger studies are needed to determine the optimal radiation protocol and to better define its impact on local recurrence and survival.

Tumor burden emerged as an important factor associated with outcome. LPR ocurred predominantly in dogs with macroscopic disease, whereas only one recurrence was documented among dogs treated for microscopic disease. Dogs undergoing surgery prior to RT had significantly longer TTP and MST on Kaplan–Meier analysis, consistent with prior reports demonstrating worse outcomes in dogs with greater tumor burden (4, 5). Surgery was also associated with a lower hazard of LPR in both univariable and exploratory multivariable Cox analyses, although these associations did not reach statistical significance. Similarly, surgery was associated with improved survival in univariable Cox analysis but was not independently associated with survival after multivariable adjustment. These findings should therefore be interpreted cautiously and may partly reflect selection bias, as surgically treated dogs generally had smaller, more resectable tumors; in the present study, their median tumor size was <4 cm compared with >6 cm in dogs treated with RT for macroscopic disease.

Although margin status was available for only a subset of dogs, the low rate of LPR among dogs with known incomplete or narrow margins suggests that hypofractionated RT combined with chemotherapy may provide effective control of microscopic residual disease. This observation is consistent with the findings of Bulakowski et al. (4), who reported relatively low rates of local recurrence following surgery and adjuvant treatment, despite the highly infiltrative nature of soft tissue HSA. However, direct comparisons are limited because the margin status of dogs that experienced local recurrence was not reported in that study. Therefore, while the present results support a potential role for RT in controlling microscopic disease, larger studies with more complete surgical margin assessment are needed to better define its impact on local tumor control.

Metastatic disease, larger tumor size, and anemia were associated with worse outcomes, consistent with prior reports and their potential role as markers of greater disease burden (1, 2, 4, 6, 7). Although metastasis and tumor size were not independently associated with survival after multivariable adjustment, metastasis remained associated with increased risk of LPR, while anemia remained independently associated with decreased survival. The higher prevalence of anemia among dogs with metastatic disease further suggests that anemia may serve as a surrogate marker of advanced disease.

Intramuscular involvement was not associated with survival in the present study, consistent with the findings of Shiu et al. (5), suggesting that disease burden and adequacy of local control may be more important determinants of outcome than anatomic location alone. In contrast, Bulakowski et al. (4) reported shorter survival times for dogs with IM tumors. One possible explanation for this discrepancy is that IM tumors may be detected later in their clinical course because they are less readily visible or palpable than SC masses, potentially resulting in larger tumor size, more extensive local invasion, or a greater likelihood of occult metastatic disease at the time of diagnosis. Given that tumor size has been identified as a prognostic factor in both the present study and previous reports, the apparent effect of tumor location observed in some studies may partially reflect differences in disease burden rather than location itself. However, the retrospective nature of available studies and the relatively small number of IM tumors evaluated preclude definitive conclusions regarding the prognostic significance of tumor location.

The relationship between initial tumor response and long-term local control should also be interpreted cautiously. Response to RT was not significantly associated with LPR or survival, indicating that early tumor shrinkage may not necessarily predict long-term outcome, consistent with the findings of Hillers et al. (6). Although dogs achieving CR or PR frequently experienced prolonged local control, these findings emphasize the distinction between objective tumor response and durable disease control. Nevertheless, the high ORR observed in dogs with macroscopic disease supports consideration of hypofractionated RT as a palliative or cytoreductive treatment option when surgery is not feasible or in advanced disease.

No significant differences in outcome were detected between the two hypofractionated RT protocols; however, interpretation is limited by the small number of dogs treated with 6 Gy × 5 fractions. These findings are consistent with those reported by Hillers et al. (6), in which hypofractionated RT achieved high objective response rates in dogs with gross nonsplenic HSA. Taken together, the results of both studies support the use of hypofractionated RT as an effective approach for local control of gross disease, while offering the practical advantages of shorter treatment duration and reduced treatment burden compared with definitive protocols.

Three dogs underwent re-irradiation months to years after initial treatment, suggesting that repeat RT may be feasible in select cases. Although outcomes could not be formally assessed, this observation raises the possibility that local recurrence may be amenable to salvage therapy when surgical excision is not feasible. Prospective studies are needed to better define cumulative dose constraints, toxicity profiles, and the clinical benefit of re-irradiation.

Chemotherapy was not associated with LPR or survival in the present study; however, interpretation of its efficacy is limited by the high rate of chemotherapy use, lack of a standardized treatment protocol, small number of dogs not receiving chemotherapy, and absence of a control group. While most dogs received doxorubicin concurrently with RT, any potential radiosensitizing effect remains speculative. However, objective responses were also observed in dogs that were not receiving doxorubicin concurrently with RT. Notably, four dogs had received doxorubicin before RT and had either failed to respond or were no longer responding when RT was initiated; all four subsequently achieved an objective response following RT. This observation supports a contribution of RT to tumor response in these dogs, although the small number of cases and retrospective study design preclude conclusions regarding the independent effect of RT or a potential interaction between RT and chemotherapy. Because response assessments were not performed at standardized time points, the influence of concurrent doxorubicin on response timing could not be reliably evaluated.

Importantly, the combination was generally well tolerated, with most radiation-associated adverse events being mild and the only grade 4 toxicity having no documented adverse impact on the patient’s quality of life. Chemotherapy-associated toxicities were generally manageable, supporting the tolerability of this multimodal treatment approach. Although a survival benefit of chemotherapy could not be demonstrated in this study, doxorubicin-based protocols have been associated with improved outcomes in dogs with visceral HSA, particularly splenic HSA, and remain a cornerstone of treatment because of the high metastatic potential of this disease (13–15). Therefore, the lack of a detectable effect in the present study should not be interpreted as evidence of a lack of benefit, but rather as a reflection of study design and limited ability to independently evaluate chemotherapy efficacy.

Limitations of this study include its retrospective design, variability in treatment selection, and inherent selection bias. In addition, several clinical and disease characteristics included in the multivariable analyses were likely interrelated. For example, dogs with larger tumors, macroscopic disease, or metastatic disease may have been less likely to undergo surgery, resulting in potential confounding among tumor burden, disease stage, and treatment selection. Consequently, the absence of an independent association for variables such as surgery, tumor size, and metastasis after multivariable adjustment should not be interpreted as evidence that these factors are not clinically relevant. Rather, their individual effects may be difficult to distinguish in this retrospective cohort, particularly given the limited number of events and relatively small sample size. Although the two hypofractionated protocols had broadly comparable calculated BED values, BED does not account for differences in overall treatment time. The 8 Gy × 3 protocol was delivered over 21 days, whereas the 6 Gy × 5 protocol was delivered every other day over a substantially shorter interval; therefore, potential differences in tumor repopulation and time-dependent normal-tissue effects are not reflected by BED comparison alone. Tumor response and progression were also assessed retrospectively and at non-standardized time points, primarily through serial clinical examinations and measurements rather than uniform cross-sectional imaging. Consequently, RECIST classification may have been less precise, particularly for small or deep changes in tumor dimensions, and time to initial and maximal response could not be reliably determined. Finally, missing data reduced cases available for multivariable analysis, and non-standardized management at progression may have influenced survival outcomes.

In conclusion, hypofractionated RT was associated with a high ORR and may provide durable local control in dogs with SC and IM HSA. LPR occurred predominantly in dogs treated for macroscopic disease, whereas recurrence was uncommon following surgery and RT for microscopic disease. Surgery was associated with improved local control and survival in unadjusted analyses, although potential selection bias should be considered. Metastatic disease and tumor size were associated with adverse outcomes, while anemia remained independently associated with decreased survival and metastasis with increased hazard of LPR in multivariable analyses. Taken together, the present findings suggest that hypofractionated RT may have a role in managing microscopic disease following surgery; however, the small number of dogs in this subgroup and potential differences in disease characteristics and treatment selection limit definitive conclusions regarding its benefit.

These findings support further investigation of multimodal treatment strategies incorporating surgery, hypofractionated RT, and systemic therapy. Prospective studies, ideally including dogs with more consistent disease characteristics and stage, are warranted to better define and confirm the potential benefit of hypofractionated RT in the early-stage or microscopic disease setting, as well as to further characterize prognostic factors for local control and survival.

## Data Availability

The original contributions presented in the study are included in the article/supplementary material, further inquiries can be directed to the corresponding author.
